# Priority clinic access or outreach to provide Sexual and Reproductive healthcare for people with mental illness?

**DOI:** 10.1192/bjo.2021.908

**Published:** 2021-06-18

**Authors:** Elizabeth Rose, Elana Covshoff, Rudiger Pittrof, Usha Kumar, Elizabeth Rose

**Affiliations:** 1 South London and Maudsley NHS Foundation Trust; 2 Guy's and St Thomas NHS Foundation Trust; 3 Kings College Hospital NHS Foundation Trust; 4 South London and Maudsley NHS Foundation Trust

## Abstract

**Aims:**

To compare two sexual and reproductive health (SRH) clinical pathways (a priority appointment at a mainstream SRH clinic versus assertive community outreach), and to explore how each improves access to care for people with psychotic mental illness, severe addictions and/or learning disability.

**Method:**

Observational, descriptive study of two clinical access pathways within SHRINE (Sexual and Reproductive Health Rights, Inclusion and Empowerment), a specialist SRH programme to improve SRH care for severely marginalised people.

The SHRINE programme delivers effective, ethical, accessible and user-centred SRH care for people with severe addiction, serious mental illness and/or learning disability in the deprived inner London boroughs of Lambeth and Southwark. These individuals often find accessing conventional SRH clinics very difficult. SHRINE clients can self-refer but most of them are referred by their health or social worker.

Clients or referrers indicate their preferred pathway: priority appointment at the mainstream clinic or assertive community outreach. The priority appointment pathway at Camberwell Sexual Health Centre (CSHC) is as flexible as possible, with minimal waiting times, reminders, invitation to bring a friend or care worker and active follow-up of non-attenders via key workers. Assertive community outreach can be in an addiction clinic, postnatal ward, mental health centre, psychiatric ward, outpatient clinic, homeless hostel or the client's home.

Time allocation for outreach and priority appointment-based care was 8 and 4 hours per week respectively. Care in both pathways was provided by senior doctors. Content of care was similar but facility for provision of gynaecological care including cervical smears and investigations for abnormal uterine bleeding e.g. pelvic ultrasound scans and endometrial biopsies were only available in the mainstream clinic setting at CSHC.

**Result:**

From May 2016 to December 2020 SHRINE received 1367 referrals from 125+ teams. We offered 1591 first or follow-up appointments of which 1369 (86%) were attended. A total of 1153 (84%) of our patient contacts occurred in the outreach setting where 93% the appointments were attended. Of the 358 appointments at CSHC 316 (60%) were attended.

**Conclusion:**

Making clinic access as simple and convenient as possible is not a sufficient strategy to meet the SRH needs of marginalised people. To enable them to realise their human right to sexual and reproductive health we need to leave our clinics and meet our clients where they are. A combined model of outreach and priority access clinic pathways is essential for provision of SRH care for people with mental illness.

